# Managing Drug‐Resistant Epilepsy and Severe Feeding Difficulties in Miller–Dieker Syndrome: The Role of Enteral Ketogenic Diet

**DOI:** 10.1155/crpe/8660691

**Published:** 2026-09-18

**Authors:** Perrone Michela, Porro Matteo, Piemontese Pasqua, Roggero Paola, Gangi Silvana, Fumagalli Monica

**Affiliations:** ^1^ Neonatal Intensive Care Unit Milan, Fondazione IRCCS Ca’ Granda Ospedale Maggiore Policlinico, Milan, Italy, policlinico.mi.it; ^2^ Paediatric Physical Medicine & Rehabilitation Service, Neonatal Intensive Care Unit Milan, Fondazione IRCCS Ca’ Granda Ospedale Maggiore Policlinico, Milan, Italy, policlinico.mi.it; ^3^ Department of Clinical Sciences and Community Health Milan, University of Milan, Lombardy, Milan, Italy, unimi.it

**Keywords:** developmental disability, ketogenic diet, pediatric, seizures

## Abstract

Miller–Dieker syndrome (MDS) is a rare genetic disorder characterized by classical lissencephaly, severe neurodevelopmental impairment, and early‐onset drug‐resistant epilepsy. Feeding difficulties and aspiration pneumonia significantly contribute to morbidity and mortality. Evidence regarding ketogenic diet (KD) therapy in patients with severe neurological impairment requiring exclusive enteral nutrition is limited. We report the case of an infant with MDS and pharmacoresistant epilepsy complicated by severe feeding intolerance and recurrent aspiration pneumonia. KD was initiated via postpyloric enteral feeding and subsequently administered through percutaneous endoscopic jejunostomy using a low ketogenic ratio (1.25:1). The diet was well tolerated and associated with seizure freedom maintained until the child’s death from an unrelated surgical complication (volvulus), improved nutritional status, and several months of clinical stability. This case highlights the feasibility of enteral KD therapy as part of a multidisciplinary, palliative‐oriented approach for managing drug‐resistant epilepsy in children with MDS.

## 1. Introduction

Miller–Dieker syndrome (MDS) is a rare gene deletion syndrome involving chromosome 17p13.3 and represents one of the most severe forms of classical lissencephaly [1, 2].

The syndrome results from a contiguous gene deletion of the 17p13.3 critical region: haploinsufficiency of *PAFAH1B1* (LIS1) is primarily responsible for the lissencephaly phenotype, while additional loss of *YWHAE* is associated with more severe cortical malformation and characteristic craniofacial dysmorphism, and *CRK* haploinsufficiency has been implicated in postnatal growth restriction [3].

The clinical phenotype includes profound developmental delay, hypotonia, distinctive craniofacial features, and early‐onset epilepsy, which is frequently refractory to antiepileptic drug therapy. Seizures often begin in the neonatal period and may evolve into epileptic spasms or other severe epileptic encephalopathies [4].

Feeding impairment is highly prevalent in MDS due to oropharyngeal dysphagia, gastroesophageal reflux, and impaired airway protection, leading to malnutrition and recurrent aspiration pneumonia. Nutritional management, therefore, plays a critical role in overall care [2, 4].

The ketogenic diet (KD) is an established nonpharmacological treatment for drug‐resistant epilepsy [5–7].

Its anticonvulsant effect is thought to result from multiple, only partially elucidated metabolic and neuromodulatory mechanisms: ketone bodies (β‐hydroxybutyrate and acetoacetate) serve as an alternative cerebral fuel source, promote neuronal membrane hyperpolarization via activation of ATP‐sensitive potassium channels, and shift the excitatory/inhibitory balance toward increased GABAergic relative to glutamatergic neurotransmission, with additional proposed contributions from enhanced mitochondrial function and modulation of the gut microbiome [8]. KD has been most extensively studied in Dravet syndrome, infantile spasms/West syndrome, Lennox–Gastaut syndrome, tuberous sclerosis complex, and GLUT1‐deficiency syndrome and more recently in broader cohorts of children with genetic epilepsy syndromes.

While KD has demonstrated efficacy across various pediatric epilepsy syndromes, its application in children with severe neurological impairment, exclusive enteral feeding, and life‐limiting conditions remains underreported [9, 10].

We present a case illustrating the role of enteral KD therapy in an infant with MDS, focusing on neurological outcomes and clinical feasibility.

## 2. Case Report

The patient was a male infant with a prenatal diagnosis of MDS, confirmed postnatally by brain magnetic resonance imaging demonstrating type 1 lissencephaly and by genetic testing revealing a 17p13.3 deletion. Seizures began within the first weeks of life and progressively worsened despite treatment with multiple antiepileptic medications, including sodium valproate, levetiracetam, and vigabatrin, uptitrated to a maximum dose of 94.3 mg/kg/day.

At 5 months of age, the patient was referred for evaluation of severe failure to thrive. Neurological examination showed marked axial hypotonia and absent postural control. Oral feeding was ineffective because of poor coordination of sucking and swallowing. Initial nutritional strategies included consistence‐modified oral feeding; however, progressive feeding intolerance, frequent vomiting, and recurrent aspiration pneumonia necessitated exclusive enteral nutrition.

Nasogastric feeding was poorly tolerated, and postpyloric enteral feeding was initiated to reduce aspiration risk. Despite optimization of feeding modality, the patient continued to experience severe drug‐resistant seizures. Following multidisciplinary discussion involving pediatric neurology, clinical nutrition, gastroenterology, and the family, KD therapy was proposed with palliative intent, aiming to reduce seizure burden and improve quality of life.

KD was initiated using a commercially available ketogenic formula delivered via postpyloric feeding, with gradual escalation to a ketogenic ratio of 1.25:1 (grams of fat to combined grams of protein and carbohydrate), initiated in March 2023 at 12 months of age. Biochemical monitoring demonstrated adequate ketosis without significant metabolic complications. Due to the need for long‐term enteral nutrition, a percutaneous endoscopic gastrostomy with jejunal extension was subsequently placed.

After the initiation of KD, seizure frequency decreased and the patient remained seizure free from the start of the diet until death, with reduced clustering and improved caregiver‐perceived alertness prior to seizure cessation. Nutritional tolerance improved, with progressive weight gain and catchup growth (Table 1). The patient experienced several months of relative clinical stability, with reduced hospitalizations, until he died at 2 years of age due to an intestinal volvulus, a surgical complication unrelated to the underlying neurological or nutritional condition; of note, the seizure‐free period was maintained up to that time. Pediatric palliative care was integrated early to support symptom management, family counseling, and shared decision‐making.

**TABLE 1:** Weight and length measurements over time.

Date	Weight (g)	Length (cm)	Notes
25 Jul 2022	4550	57.5	< 3rd percentile WHO
18 Aug 2022	4800	58	< 3rd percentile WHO
08 Jun 2023	10,025	—	Hospital admission (Vidas hospice)
24 Jul 2023	10,860	—	Hospital discharge (Vidas hospice)
12 Sep 2023	10,600	76	—
13 Nov 2023	10,430	77	—
23 Apr 2024	10,700	79.5	—

## 3. Discussion

Children with MDS present complex therapeutic challenges due to the coexistence of severe epileptic encephalopathy and feeding impairment. This case demonstrates that enteral KD therapy can be safely implemented even in patients requiring jejunal feeding, provided that careful metabolic and nutritional monitoring is ensured.

A relatively low ketogenic ratio was chosen to balance seizure control and tolerability, consistent with a palliative‐oriented approach. Seizure freedom was maintained for the remainder of the patient’s life, and the intervention contributed to clinical stability and improved nutritional status, outcomes that are particularly meaningful in the context of life‐limiting neurological disease [11, 12].

Notably, seizure control was not lost over time: the patient remained seizure free from KD initiation until death, which resulted from an unrelated surgical complication (intestinal volvulus) rather than from recurrence of epilepsy or progression of the underlying neurological disease. The apparent limited duration of benefit, therefore, reflects the length of survival rather than a waning therapeutic effect although the absence of a control period and the inherent limitations of a single case preclude definitive conclusions regarding causality.

Reports of KD administered via jejunal feeding in patients with profound neurodevelopmental disorders are scarce. This case adds to the limited literature supporting individualized, multidisciplinary epilepsy management strategies in children with rare genetic syndromes.

## 4. Conclusion

Enteral KD therapy may represent a feasible adjunctive treatment for drug‐resistant epilepsy in selected patients with MDS and severe feeding difficulties. In the context of advanced neurological disease, KD should be considered within a multidisciplinary and palliative care framework, with emphasis on seizure burden reduction, nutritional stability, and quality of life.

## Author Contributions

All authors contributed to the conception, clinical management, drafting, and revision of the manuscript.

## Funding

This study was partially funded by Italian Ministry of Health, Current research IRCCS.

## Disclosure

All authors have read and approved the final manuscript.

## Ethics Statement

Written informed consent for publication was obtained from the patient’s caregivers. Ethical standards of the institution were followed.

## Conflicts of Interest

The authors declare no conflicts of interest.

## Supporting Information

Additional supporting information can be found online in the Supporting Information section.

## Supporting information


**Supporting Information** Care checklist.

## Data Availability

The data that support the findings of this study are available on request from the corresponding author. The data are not publicly available due to privacy or ethical restrictions.
